# The genetic and epigenetic regulation of *CD55* and its pathway analysis in colon cancer

**DOI:** 10.3389/fimmu.2022.947136

**Published:** 2023-01-18

**Authors:** Jiawei Liu, Ning Fu, Zhenbang Yang, Ang Li, Hongjiao Wu, Ye Jin, Qinqin Song, Shanshan Ji, Hongxue Xu, Zhi Zhang, Xuemei Zhang

**Affiliations:** ^1^ Affiliated Tangshan Gongren Hospital, North China University of Science and Technology, Tangshan, China; ^2^ College of Life Science, North China University of Science and Technology, Tangshan, China; ^3^ School of Public Health, North China University of Science and Technology, Tangshan, China

**Keywords:** complement, *CD55*, colon cancer, *NF-κB*, MiR-27a-3p

## Abstract

**Background:**

*CD55* plays an important role in the development of colon cancer. This study aims to evaluate the expression of *CD55* in colon cancer and discover how it is regulated by transcriptional factors and miRNA.

**Methods:**

The expression of *CD55* was explored by TIMER2.0, UALCAN, and Human Protein Atlas (HPA) databases. TRANSFAC and Contra v3 were used to predict the potential binding sites of transcription factors in the *CD55* promoter. TargetScan and starBase v2.0 were used to predict the potential binding ability of miRNAs to the 3′ untranslated region (3′UTR) of *CD55*. SurvivalMeth was used to explore the differentially methylated sites in the *CD55* promoter. Western blotting was used to detect the expression of *TFCP2* and *CD55*. Dual-luciferase reporter assay and chromatin immunoprecipitation (ChIP) assay were performed to determine the targeting relationship of *TFCP2*, *NF-κB*, or miR-27a-3p with *CD55*. *CD55*-related genes were explored by constructing a protein–protein interaction (PPI) network and performing pathway analysis by Gene Ontology (GO) and Kyoto Encyclopedia of Genes and Genomes (KEGG).

**Results:**

*CD55* was highly expressed in colon cancer tissues. The mRNA and protein expression levels of *TFCP2* were reduced by si-*TFCP2*. *NF-κB* mRNA was obviously reduced by *NF-κB* inhibitor and increased by *NF-κB* activator. *CD55* protein was also inhibited by miR-27a-3p. Dual-luciferase reporter assays showed that after knocking down *TFCP2* or inhibiting *NF-κB*, the promoter activity of *CD55* was decreased by 21% and 70%, respectively; after activating *NF-κB*, the promoter activity of *CD55* increased by 2.3 times. As *TFCP2* or *NF-κB* binding site was mutated, the transcriptional activity of *CD55* was significantly decreased. ChIP assay showed that *TFCP2* and *NF-κB* combined to the promoter of *CD55*. The luciferase activity of *CD55* 3′UTR decreased after being co-transfected with miR-27a-3p mimics and increased by miR-27a-3p antagomir. As the miR-27a-3p binding site was mutated, we did not find any significant effect of miR-27a-3p on reporter activity. PPI network assay revealed a set of *CD55*-related genes, which included *CFP*, *CFB*, *C4A*, and *C4B.* GO and KEGG analyses revealed that the target genes occur more frequently in immune-related pathways.

**Conclusion:**

Our results indicated that *CD55* is regulated by *TFCP2*, *NF-κB*, miR-27a-3p, and several immune-related genes, which in turn affects colon cancer.

## 1 Introduction

According to GLOBOCAN 2020, colorectal cancer (CRC) ranks third in terms of morbidity and mortality in the world, presenting a serious threat to human health. Also, over the past decade, the rate of decline in CRC mortality has slowed (1). The incidence of CRC is known to be significantly influenced by early-life exposures (2). Thus, it is essential to find effective molecular targets for colon cancer treatment.

Gene expression is a highly regulated process. Given the complexity of gene regulation, it is not surprising that many human diseases, including various cancers, are caused by defective gene regulation. Transcription factors (TFs) are known to regulate chromatin and transcription by recognizing specific DNA sequences and subsequently forming a complex (3). MicroRNAs (miRNAs) are endogenous, and small non-coding RNAs are implicated in nearly all known physiological and pathological processes, such as cell proliferation, differentiation, and apoptosis. Importantly, miRNAs act as important regulators of several genes at the post-transcriptional level in almost all kinds of cancer cells. Multiple studies have reported that miRNAs exhibit aberrant expression in a variety of cancers.

DNA methylation is an epigenetic mechanism that tends to be crucial in the regulation of gene expression (4–6). Gene expression can be affected by the bonding of the methyl group to the cytosine nucleotide. DNA methylation in the promoter region of genes is often associated with the repression of transcription (7). The DNA methylation level in tumor tissues is mostly reported to be lower than that in normal adjacent tissues. During the development of cancer, the level of hypomethylation of genomic DNA increases as lesions progress from benign cell proliferation to aggressive cancer (8).

The complement system is a crucial element of innate immunity, and it also plays an essential role in the regulation of adaptive immunity (9). The activation of the complement system promotes cell proliferation in various cancers (10). *CD55*, also known as decay-accelerating factor (*DAF*), is one of the membrane-bound complement regulatory proteins (*mCRP*s) that play a key role in maintaining the homeostasis of the complement system. Dho and his colleagues found that *CD55* was over-expressed in metastatic colon cancer tissues, and inhibition of *CD55* could restrain colon cancer tumorigenesis and metastasis (11). The high expression of *CD55* has a significantly decreased 7-year survival rate for colon cancer (12). Comprehensive bioinformatics analysis has also shown that *CD55* was positively correlated with infiltration levels of CD8^+^ T cells, neutrophils, and dendritic cells in colon cancer, suggesting its potential role in tumor immune regulation (13).

Since *CD55* might be used as a prognostic indicator and a therapeutic target in colon cancer, we investigated the possible mechanism of the regulation of *CD55* in colon cancer and its impact on the molecular and immunological characteristics of colon cancer.

## 2 Materials and methods

### 2.1 The expression analysis of *CD55* and miR-27a

TIMER2.0 (http://timer.cistrome.org/) is a web server freely available to the research community. It consists of three main components: immunization, exploration, and estimation. The “Diff Exp” component allows users to compare gene expression differences between multiple tumor and normal tissues. In this study, we used TIMER2.0 to analyze the expression difference of *CD55* between cancer tissues and normal tissues in a pan-cancer way. We analyzed the expression level of *CD55* in colorectal cancer and its association with different histopathological types by UALCAN (http://ualcan.path.uab.edu), which contains cancer OMICS data. The protein expression of *CD55* in human normal colon tissue and colon cancer tissue was verified by Human Protein Atlas (HPA; https://www.proteinatlas.org/). The HPA database, an antibody-based approach combined with transcriptomic data to outline global expression profiles, is the largest and most comprehensive public database of the spatial distribution of proteins in human tissues and cells (14). We downloaded the available miRNA data from the Gene Expression Omnibus (GEO) database (GSE48267 and GSE59856). We used the Limma program to analyze the expression of miR-27a in colorectal cancer.

### 2.2 *In silico* analysis of *CD55* regulatory region

From *CD55*, 2 kb of 5′ flanking region and 3′ untranslated region (3′UTR) was downloaded from the National Center for Biotechnology Information (NCBI) database. Transcription factor binding sites in the promoter of *CD55* were predicted by using the TRANSFAC program (http://gene-regulation.com) and ConTra v3 online web tool (http://bioit2.irc.ugent.be/contra/v3/). “Vertebrates” was chosen as “Matrix groups”, and “cut-offs” was set to “minimize the sum of both error rates”. The potential miRNA binding sites in 3′UTR of *CD55* were screened using TargetScanHuman 7.2 (https://www.targetscan.org/vert_72/) and starBase v2.0 (https://starbase.sysu.edu.cn/starbase2/index.php).

### 2.3 Cell lines and reagents

Human colon cancer cells HCT-116 and LOVO were provided by Procell (Wuhan, China). All plasmids (pGL3-Basic, pRL-SV40, and psiCHECK-2 plasmid) were purchased from Promega (Madison, WI, USA). The si-*TFCP2*, miR-27a-3p mimics, and miR-27a-3p antagomir were synthesized by GenePharma (Shanghai, China). The sequences of si-*TFCP2* were 5′-GCU AAU CCA ACU CAA CUA ATT-3′ and 5′-UUA GUU GAG UUG GAU UAG CTT-3′. The sequences of siRNA control were 5′-UUC UCC GAA CGU GUC ACG UTT-3′ and 5′-ACG UGA CAC GUU CGG AGA ATT-3′. The sequences of miR-27a-3p mimics were 5′-UUC ACA GUG GCU AAG UUC CGC-3′ and 5′-GGA ACU UAG CCA CUG UGA AUU-3′. The sequences of mimics control were 5′-UUC UCC GAA CGU GUC ACG UTT-3′ and 5′-ACG UGA CAC GUU CGG AGA ATT-3′. The sequence of miR-27a-3p antagomir was 5′-GCG GAA CUU AGC CAC UGU GAA-3′. The sequence of antagomir control was 5′-CAG UAC UUU UGU GUA CAA-3′. *NF-κB* inhibitor (B5556) and *NF-κB* activator (TNFα) (HZ-1014) were purchased from Sigma-Aldrich (New Jersey, USA) and ProteinTech (Chicago, USA), respectively.

### 2.4 Construction of *CD55* promoter and 3′UTR plasmid

The primer pair used to amplify the promoter of *CD55* (1,963 bp) was *CD55*-PF (5′-GG GGTACC CCT CTC TAT GAA GGG CA-3′)/*CD55*-PR (5′-CCC AAGCTT GGG GAC GGC GGG AAC CAC GAC-3′) with protective bases and the recognition sites (underlined bases) of *Kpn*I or *Hin*dIII in each primer. This PCR product was then cloned into a pGL3-Basic plasmid. The PCR primers to amplify the fragment of 3′UTR (1,237 bp) were *CD55*_3′UTR_-PF (5′-CCG CTCGAG TGC CTT CAT TTA GGA TGC TTT CA-3′) and *CD55*_3′UTR_-PR (5′-GTAA GCGGCCGC TTT ACA GTG AAA TGC CAT GAA CG-3′), of which protective bases and recognition sites (underlined) of *Xho*I and *Not*I sites were added. The 3′UTR PCR product was then cloned into a psiCHECK-2 plasmid. Successful plasmids were then designed wild-type plasmids as pGL3-*CD55_pro_*-Wt and psi-*CD55*_3′UTR_-Wt. The *CD55* promoter fragment containing *TFCP2* or *NF-κB* binding sites was synthesized to generate mutant type (pGL3-*CD55*_pro_-*TFCP2*-Mut and pGL3-*CD55*_pro_-*NF-κB*-Mut) by Sangon Biotech (Shanghai, China). The *CD55* 3′UTR fragment containing miR-27a-3p binding sites was also synthesized to generate a mutant type (psi-*CD55*_3′UTR_-Mut).

### 2.5 Cell culture and treatment

Human colon cancer cells were cultured in complete Dulbecco’s modified Eagle’s medium (DMEM; Thermo Fisher Scientific, Waltham, MA, USA) with 10% fetal bovine serum (FBS; Thermo Fisher Scientific, USA), 1% penicillin–streptomycin (100 U/ml of penicillin and 100 mg/ml of streptomycin, P&S; Thermo Fisher Scientific, USA) in an atmosphere with 5% CO_2_ at 37°C. Cells were transfected with Lipofectamine 2000 reagent (Thermo Fisher Scientific, USA), according to the manufacturer’s instructions. For *TFCP2* binding analysis, 500 ng of pGL3-*CD55*_pro_/pGL3-*CD55*_mut_ plasmid and 0.5 ng of pRL-SV40 plasmid were co-transfected with si-*TFCP2* or negative control into HCT116 cells for 24 h. For *NF-κB* binding analysis, colon cancer cells were transfected with 500 ng of pGL3-*CD55*_pro_/pGL3-*CD55*_mut_ and 0.5 ng of pRL-SV40 for 24 h and then treated with or without 20 μM of *NF-κB* inhibitor/50 ng of *NF-κB* activator for another 24 h. For miRNA binding analysis, 30 ng of psi-*CD55*_3′UTR_-Wt/psi-*CD55*_3′UTR_-Mut was co-transfected with 100 nM of miR-27a-3p mimics/control or 20 nM of miR-27a-3p antagomir/control into HCT116 cells for 24 h.

### 2.6 cDNA synthesis and qRT-PCR

Total RNA was extracted from HCT116 cells using TRIzol reagent (Thermo Fisher Scientific, USA) and was then reversely transcribed into cDNA with RevertAid First Strand cDNA Synthesis Kit (Thermo Fisher Scientific, USA). *TFCP2* mRNA was detected using Power SYBR Green PCR Master Mix (Thermo Fisher Scientific, USA) in ABIPRISM^®^ 7900HT Fast Real-Time PCR System (Applied Biosystems, Foster City, CA, USA). The amplification procedure was 50°C for 2 min and 95°C for 2 min, followed by 45 cycles of 95°C for 15 s and 60°C for 2 min. *GAPDH* was used as the reference gene. The primer pairs for the amplification of *TFCP2*, *NF-κB*, and *GAPDH* were *TFCP2*-PF/*TFCP2*-PR (5′-TCA CGT ATG TCA ATA ACT CCC CA-3′/5′-GTG TGG TTG GTA AGA GGT T-3′), *NF-κB-*PF/*NF-κB-*PR (5′-AAC AGA GAG GAT TTC GTT TCC G-3′/5′-TTT GAC CTG AGG GTA AGA CTT CT-3′), and *GAPDH*-PF/*GAPDH*-PR (5′-ACA ACT TTG GTA TCG TGG AAG G-3′/5′-GCC CAT CAC GCC ACA GTT TC-3′). Three repetitions were performed for each reaction. The relative mRNA expression was analyzed using the 2^−ΔΔCt^ method.

### 2.7 Dual-luciferase reporter assay

Colon cancer cells were plated at 2 × 10^5^ cells per well into 24-well plates. When cells reached 70%–80% confluence, each plasmid was then transfected into cells. After 24 h, cells were collected, and luciferase and Renilla reporter signals were detected using the Dual-luciferase Reporter Assay System (Promega, USA) by GloMax^®^ 20/20 Luminometer (Promega, USA).

### 2.8 Western blotting analysis

The colon cancer cells were lysed with radioimmunoprecipitation assay (RIPA) buffer (Thermo Fisher Scientific, USA). After being quantified and denatured, samples were separated by 10% sodium dodecyl sulfate–polyacrylamide gel electrophoresis (SDS-PAGE) and then transferred to a polyvinylidene difluoride (PVDF) membrane (Millipore, Billerica, MA, USA). After being blocked for 1 h with 5% skimmed milk in TBST at room temperature, the membrane was incubated with the primary antibody overnight at 4°C and then washed and incubated with horseradish peroxidase (HRP)-conjugated secondary antibody for 1 h at room temperature. Specific protein was then developed by using enhanced chemiluminescence (ECL) luminescence reagents (Amersham, UK). The densitometry analysis was performed using ImageJ (National Institutes of Health, USA). *β*-Actin was applied as a reference control. The following primary antibodies were used: anti−*CD55* (1:10,000 dilution; ab133684; Abcam, Cambridge, UK), anti−*NF-κB p*65 (1:5,000 dilution; ab32536; Abcam), and anti−*TFCP2* (1:5,000 dilution; 15203-1-AP; ProteinTech).

### 2.9 Chromatin immunoprecipitation assay

LOVO cells (2 × 10^6^) were plated in a 6-mm dish for the chromatin immunoprecipitation (ChIP) experiment by using a ChIP Assay kit (Thermo Fisher Scientific, USA) following the manufacturer’s protocol. To verify if *NF-κB* or *TFCP2* binds to the promoter of *CD55*, a total of 5 μg of sheared DNA was used for chromatin immunoprecipitation using anti-*NF-κB* (1:250 dilution; ab32536; Abcam) or anti−*TFCP2* antibody (1:250 dilution; 15203-1-AP; ProteinTech). The immunoprecipitated DNA was then amplified using specific primers to analyze the transcription factor binding site of *CD55*. The *CD55* promoter-specific primers were described as follows: *NF-κB* site-PF/*NF-κB* site-PR (5′-CGT GTG GGG TGA GTA GGG-3′/5′-ATG CTG GTG AGC GGC GAG-3′) and *TFCP2* site-PF/*TFCP2* site-PR (5′-CGT CTT GTT TGT CCC ACC C-3′/5′-GCA GTA AGC AGA AGC CTC G-3′).

### 2.10 Cell viability detection by CCK8 assay

Cell viability was analyzed by Cell Counting Kit 8 (CCK8; Dojindo, Kumamoto, Japan) according to the manufacturer’s protocol. Cells were seeded and cultured at a density of 5 × 10^3^/well into 96-well microplates. After psi-*CD55*3′UTR-Wt was co-transfected with miR-27a-3p mimics or mimics control for 24 h, 10 μl of CCK8 reagent was added and cultured for 30 mins. All experiments were performed in triplicate. The absorbance was measured at 450 nm using a microplate reader (Multiskan FC, Thermo Fisher Scientific, USA).

### 2.11 PPI network construction of *CD55*-related proteins

STRING consortium 2022 (https://string-db.org) aims to integrate all known and predicted associations between proteins, including both physical interactions and functional associations. We set the network type to “full STRING network”, the required score to “high confidence (0.700)”, and the size cutoff to “no more than 20 interactors” to obtain the *CD55*-related protein. We used the Cytoscape 3.9.1 platform to construct the protein interaction network (protein–protein interaction (PPI) network) and analyze the network characteristics.

### 2.12 GO and KEGG analyses of *CD55*-related genes

We used the “ggplot2” R package and DAVID 6.8 (http://www.david.niaid.nih.gov) database to conduct Gene Ontology (GO) and Kyoto Encyclopedia of Genes and Genomes (KEGG) analysis of *CD55*-related proteins (*p* < 0.05). We performed the visualization of GO and KEGG enrichment by R version 3.6.1.

### 2.13 The detection of methylation modification of *CD55* and its effect on the prognosis of colon cancer patients

SurvivalMeth (http://bio-bigdata.hrbmu.edu.cn/survivalmeth/) investigates the effect of DNA methylation-related functional elements on prognosis, which was developed by Harbin Medical University. We analyzed the methylation sites and their effect on the survival time of colon cancer patients of *CD55* by using SurvivalMeth.

### 2.14 Statistical analyses

All analyses were performed with R version 3.6.1 and its appropriate packages. Statistical graphs were produced in GraphPad Prism 8. Statistical significance of the Wilcoxon rank sum test or t-test was used for two-group comparisons. All statistical tests were two-sided, and *p* < 0.05 was considered to be statistically significant.

## 3 Results

### 3.1 The expression analysis of *CD55* in colorectal cancer tissues

**Figure 1:**
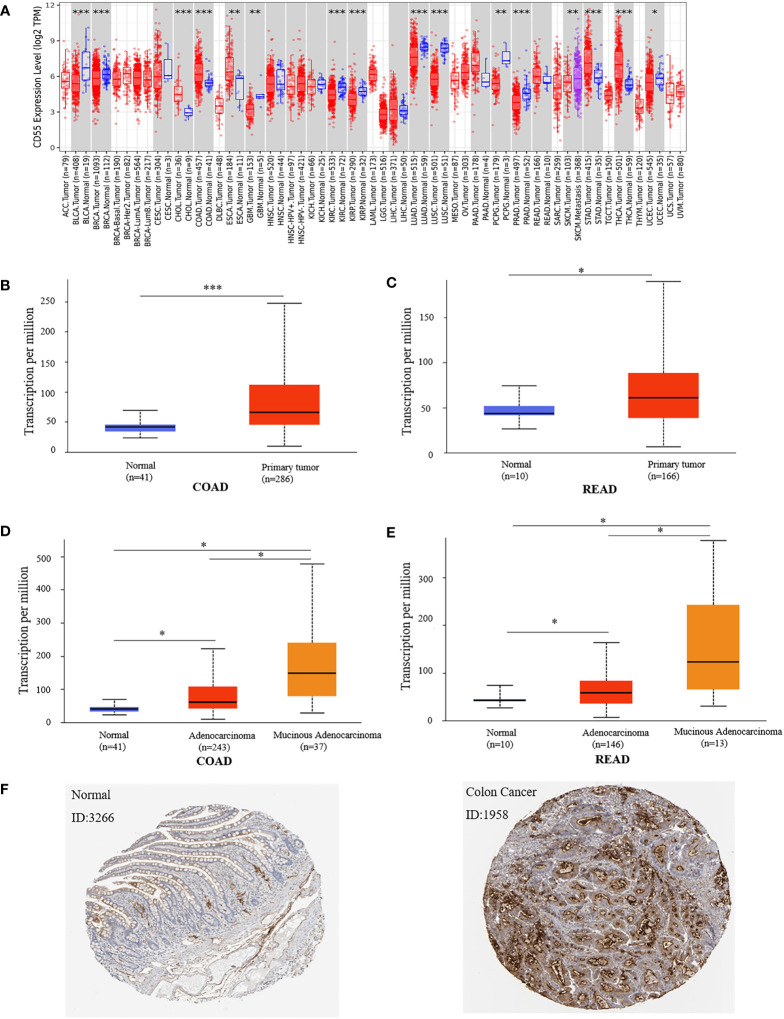
Expression of *CD55* in colon cancer tissue and adjacent normal tissue. **(A)** *CD55* expression levels in different tumor types from TCGA database were determined by TIMER2.0. **(B, C)** Quantification of *CD55* expression levels in colorectal cancer (COAD, colon adenocarcinoma; READ, rectal adenocarcinoma) and paired adjacent normal tissues. **(D, E)** Quantification of *CD55* expression in different pathological types of colorectal cancer. **(F)** Protein levels of *CD55* in colorectal cancer and normal tissues by HPA database (*p < 0.05, **p < 0.01, ***p < 0.001). TCGA, The Cancer Genome Atlas.

Using the TIMER2.0 online program, we found that *CD55* was highly expressed in a variety of solid cancers, including colorectal cancer (**Figure 1A**). There are two pathological types of colorectal cancer tissues, adenocarcinoma and mucinous adenocarcinoma. Using the UALCAN database, we evaluated the expression of *CD55* in colorectal cancer tissues and its relationship with different pathological types and found that *CD55* was highly expressed in either colorectal adenocarcinoma or mucinous adenocarcinoma when compared with that in adjacent normal tissues. Additionally, the average level of *CD55* in mucinous adenocarcinoma tissue is significantly higher than that in adenocarcinoma tissue (**Figures 1B** **–E**). Immunohistochemical images from the HPA database showed that the expression *CD55* was higher in colorectal cancer than in adjacent normal tissue (**Figure 1F**).

### 3.2 Transcriptional regulation of *CD55*

**Figure 2:**
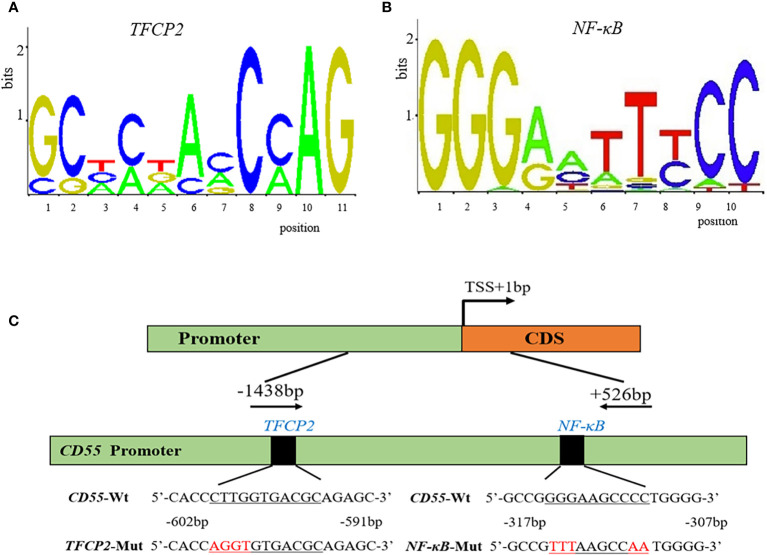
The potential binding sites and the diagram of the *CD55* promoter. **(A, B)** The potential conserved sequence of *TFCP2* and *NF-κB* binding site in the promoter of *CD55* by Contra v3. **(C)** A diagram of the *CD55* promoter region that was cloned in the luciferase reporter vector.

To predict the potential TF binding sites of *CD55*, we used TRANSFAC online program, and it generated eight transcriptional factors, including *COMP1*, *Hand1/E47*, *CDP CR1*, *Pax-4*, *TFCP2*, *Nkx2-5*, *c-Rel*, and *NF-κB* (**Table 1**). According to the core binding characteristic of these TFs provided by the Contra v3 database, we found that *TFCP2* and *NF-κB* were the most likely to bind to *CD55* (**Figures 2A** **–C**).

**Table 1:** The potential binding transcription factors of the *CD55*.

Factor name	Core match	Matrix match	Sequence
*COMP1*	1.00	0.82	ctgtagGATTGgctccagcaatgg
*Hand/E47*	1.00	0.96	ctagCCAGAcccagat
*CDP CR1*	1.00	0.93	cccaTCAATg
*Pax-4*	0.93	0.84	tgggtTGATGggtgcagcaaa
*Pax-4*	0.90	0.88	agcacTCAAGcgcggggatgc
*TFCP2*	0.95	0.9	CTTGGtgacgc
*Nkx2-5*	1.00	1.00	caTAATTa
*NF-κB*	1.00	1.00	GGGAAttccc
*c-Rel*	1.00	1.00	GGAAAttccc
*NF-κB*	1.00	1.00	gggaaGCCCC

**Figure 3:**
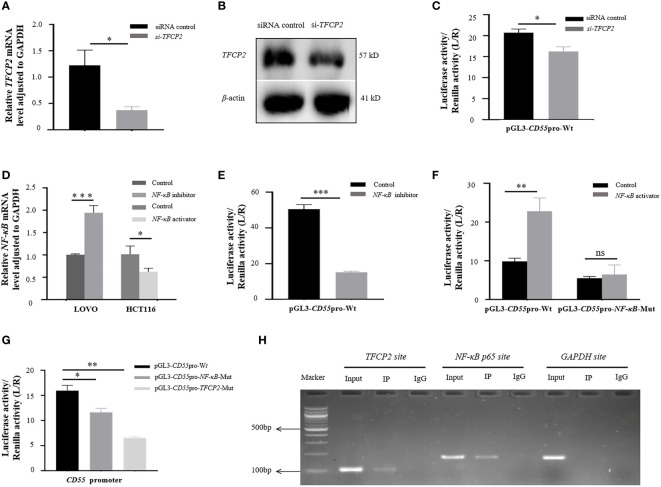
The regulation of transcription factors to the promoter of *CD55*. **(A)** The mRNA level of *TFCP2* by RT-PCR after HCT116 cells transfected with si-*TFCP2* or siRNA control for 24 h. **(B)** The protein expression of *TFCP2* by Western blotting after HCT116 cells transfected with si-*TFCP2* or siRNA control for 48 h. **(C)** Dual-luciferase assay of pGL3-*CD55*_pro_-Wt in HCT116 cells after co-transfected with si-*TFCP2.* **(D)** The mRNA level of *NF-κB* in HCT116 cells treated with *NF-κB* inhibitor and that in LOVO cells treated with *NF-κB* activator for 24 h. **(E)** The luciferase activity of pGL3-*CD55*_pro_-Wt in HCT116 cells treated with or without *NF-κB* inhibitor. **(F)** The luciferase activity of pGL3-*CD55*_pro_-Wt and pGL3-*CD55*_pro_-*NF-κB*-Mut in LOVO cells treated with or without *NF-κB* activator (TNFα). **(G)** *CD55* promoter activity assay in pGL3-*CD55*_pro_-Wt, pGL3-*CD55*_pro_-*NF-κB*-Mut, and pGL3-*CD55*_pro_-*TFCP2*-Mut plasmids. Luciferase activity was normalized to Renilla luciferase activity (L/R). **(H)** The binding of *TFCP2* and *NF-κB* to the promoter of *CD55* by ChIP. IgG from rabbits served as a control (*p < 0.05, **p < 0.01, ***p < 0.001, ns, no significance). ChIP, chromatin immunoprecipitation.

To confirm the regulation of *TFCP2* to the expression of *CD55*, we used *TFCP2* siRNA to treat HCT116 cells. We found that *TFCP2* siRNA effectively reduced the expression of *TFCP2* (**Figures 3A, B** **)**. Dual-luciferase reporter assay showed that the reporter activity of pGL3-*CD55*_pro_-Wt with *TFCP2* knockdown decreased by 21% compared with that without *TFCP2* knockdown (*p* < 0.05) (**Figure 3C**). After the *TFCP2* binding site was mutated, the promoter activity of *CD55* was reduced by 66% (**Figure 3G**).

We also evaluated the effect of *NF-κB* on the promoter activity of *CD55*. We found that the expression of *NF-κB* was significantly decreased by the *NF-κB* inhibitor and increased by the *NF-κB* activator (**Figure 3D**). The luciferase reporter analysis showed that the promoter activity of *CD55* was decreased by 70% due to the *NF-κB* inhibitor (*p* < 0.01) (**Figure 3E**). The transcriptional activity of *CD55* was activated by the *NF-κB* activator; however, after we mutated the binding site of *NF-κB*, we did not see the effect of the *NF-κB* activator on the luciferase reporter activity (**Figure 3F**). After the *NF-κB* binding site was mutated, the promoter activity of *CD55* was reduced by 42% (**Figure 3G**).

ChIP assay presented that *TFCP2* and *NF-κB* were combined to the promoter of *CD55* (**Figure 3H**), which further supported the binding capability of *TFCP2* and *NF-κB* to the promoter of *CD55*.

### 3.3 Regulation of *CD55* 3′UTR activity

**Figure 4:**
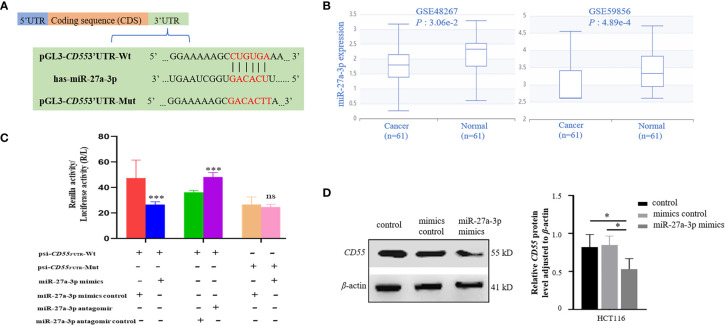
Binding and regulation of miRNA to *CD55* 3′UTR. **(A)** TargetScan and starBase v2.0 database predicted miR-27a-3p binding site in the 3′UTR of *CD55.* **(B)** The differential expression of miR-27a in two colon cancer GEO datasets (*p* < 0.05). **(C)** Verification of the potential binding of miR-27a-3p mimics or antagomir with *CD55* using luciferase reporter assay. The luciferase reporter activity of *CD55* 3′UTR reduced by miR-27a-3p mimics and increased miR-27a-3p antagomir (*p* < 0.001). The relative luciferase activity was normalized to the Renilla luciferase activity. Each experiment was performed in triplicate. Data are presented as the mean value ± SD (*p < 0.05; **p < 0.01; ***p < 0.001, ns, no significance). **(D)** The protein expression of *CD55* in HCT116 cells determined by Western blotting and densitometry analysis after cells were transfected with or without miR-27a-3p mimics (negative control) or with mimics control for 48 h (*p < 0.05). GEO, Gene Expression Omnibus.

Through TargetScan and starBase v2.0 database, we found that there was one potential binding site of miR-27a-3p in the 3′UTR of *CD55* (**Figure 4A**). Using the dataset from GEO, we found that miR-27a was downregulated in colorectal cancer (**Figure 4B**). We then conducted a CCK8 assay to see the effect of miR-27a on cell proliferation and found that miR-27a inhibited cell proliferation by 37.8%. We constructed luciferase reporter plasmid of *CD55* 3′UTR with or without mutated miR-27a-3p binding site. By co-transfecting with miR-27a-3p mimics or its mimics control into HCT116 or using an antagomir of miR-27a-3p or its antagomir control, we found that the luciferase reporter activity of *CD55* 3′UTR reduced by 56% by miR-27a-3p mimics and increased by 33% by miR-27a-3p antagomir (*p* < 0.001) (**Figure 4C**). Next, after transfecting miR-27a-3p mimics into HCT116, we found that *CD55* was upregulated by miR-27a-3p (**Figure 4D**).

### 3.4 *CD55* was regulated by methylation modification

**Figure 5:**
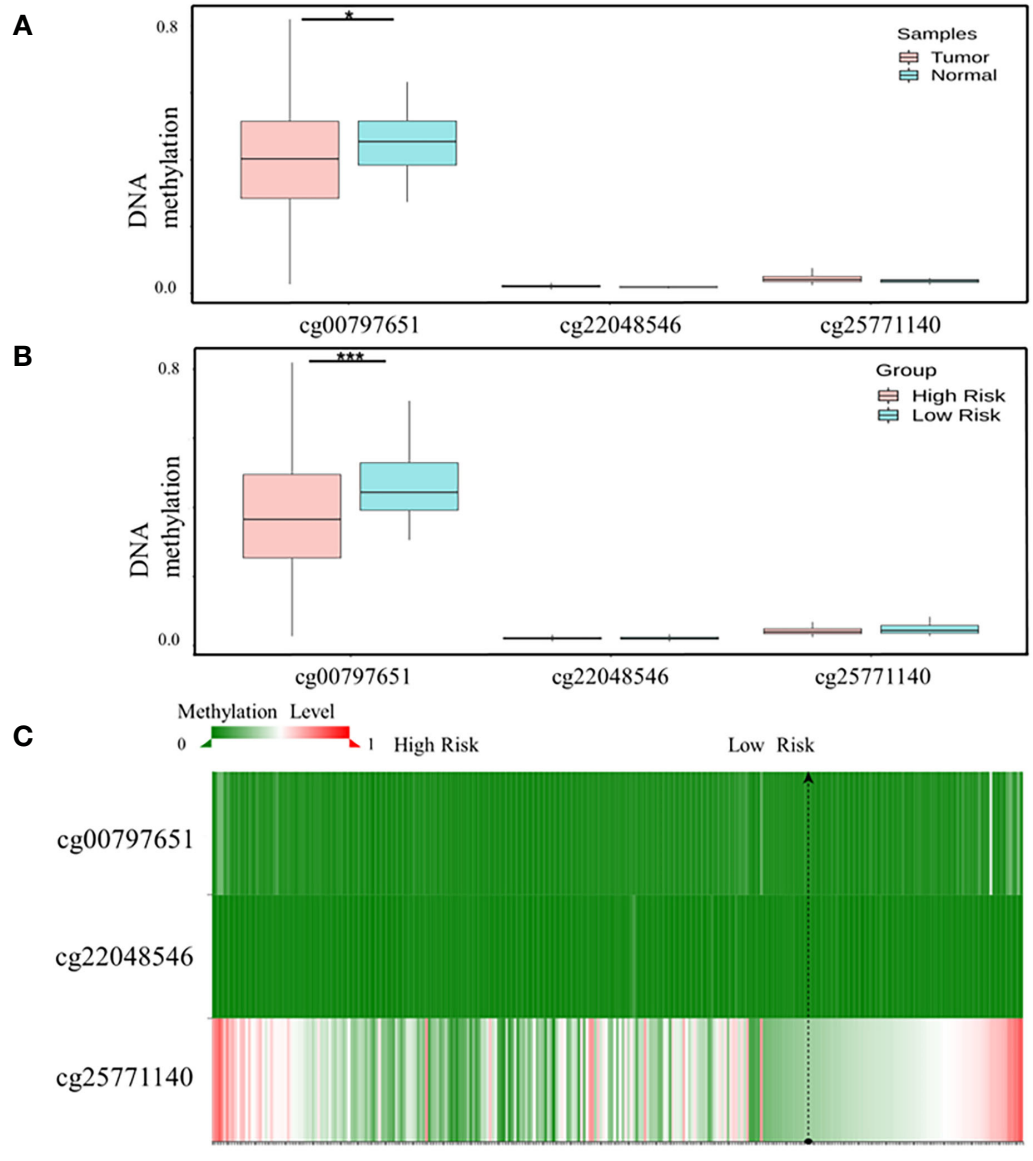
Methylation modification of *CD55* promoter in colon cancer. **(A)** The cg sites of *CD55* promoter region in COAD by SurvivalMeth. **(B)** The methylation level of CpGs in high- and low-risk groups. **(C)** The heatmap of three cg sites and their methylation level (*p < 0.05, ***p < 0.001). COAD, colon adenocarcinoma.

SurvivalMeth presented that three methylation sites (cg00797651, cg22048546, and cg25771140) appeared in the promoter of *CD55*. Among these sites, cg00797651 has a lower DNA methylation level in tumor samples than in normal samples (*p* < 0.01) (**Figure 5A**). Then, we divided the samples into high- and low-risk groups by differentially methylated sites and compared the methylation level of CpGs between the two groups. We found that the high-risk group had lower methylation levels (**Figures 5B** **, C**).

### 3.5 Protein–protein interaction network analysis of *CD55*-related proteins and their functional annotation

**Figure 6:**
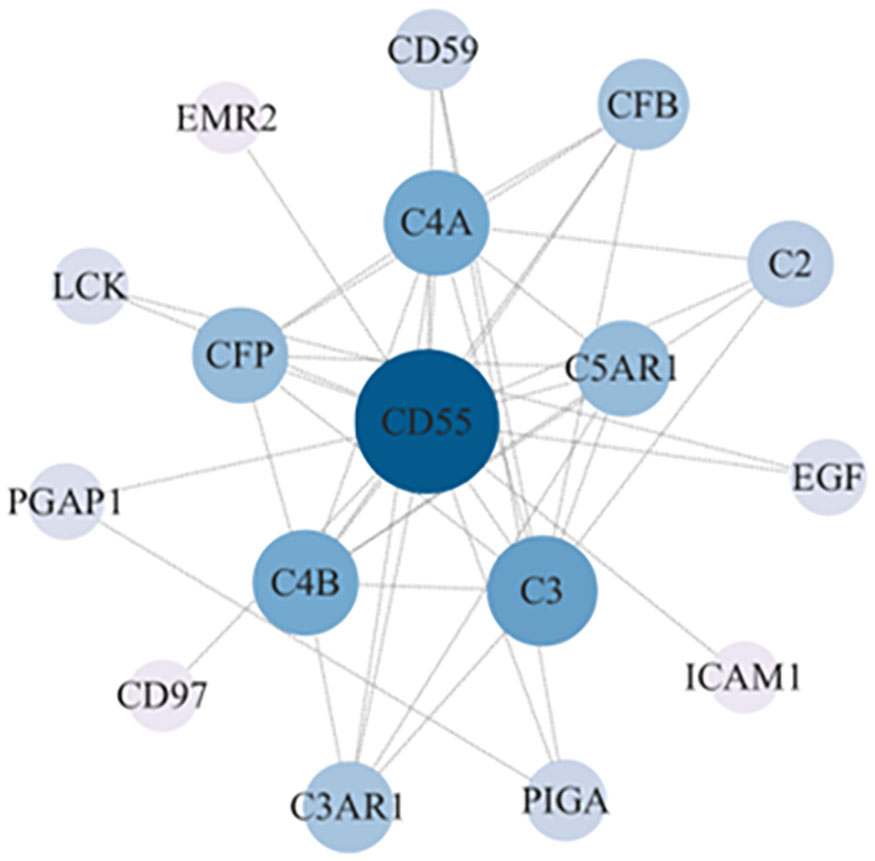
*CD55* PPI network analysis. The PPI network of *CD55* and related proteins were visualized by the STRING database. PPI, protein–protein interaction.

**Figure 7:**
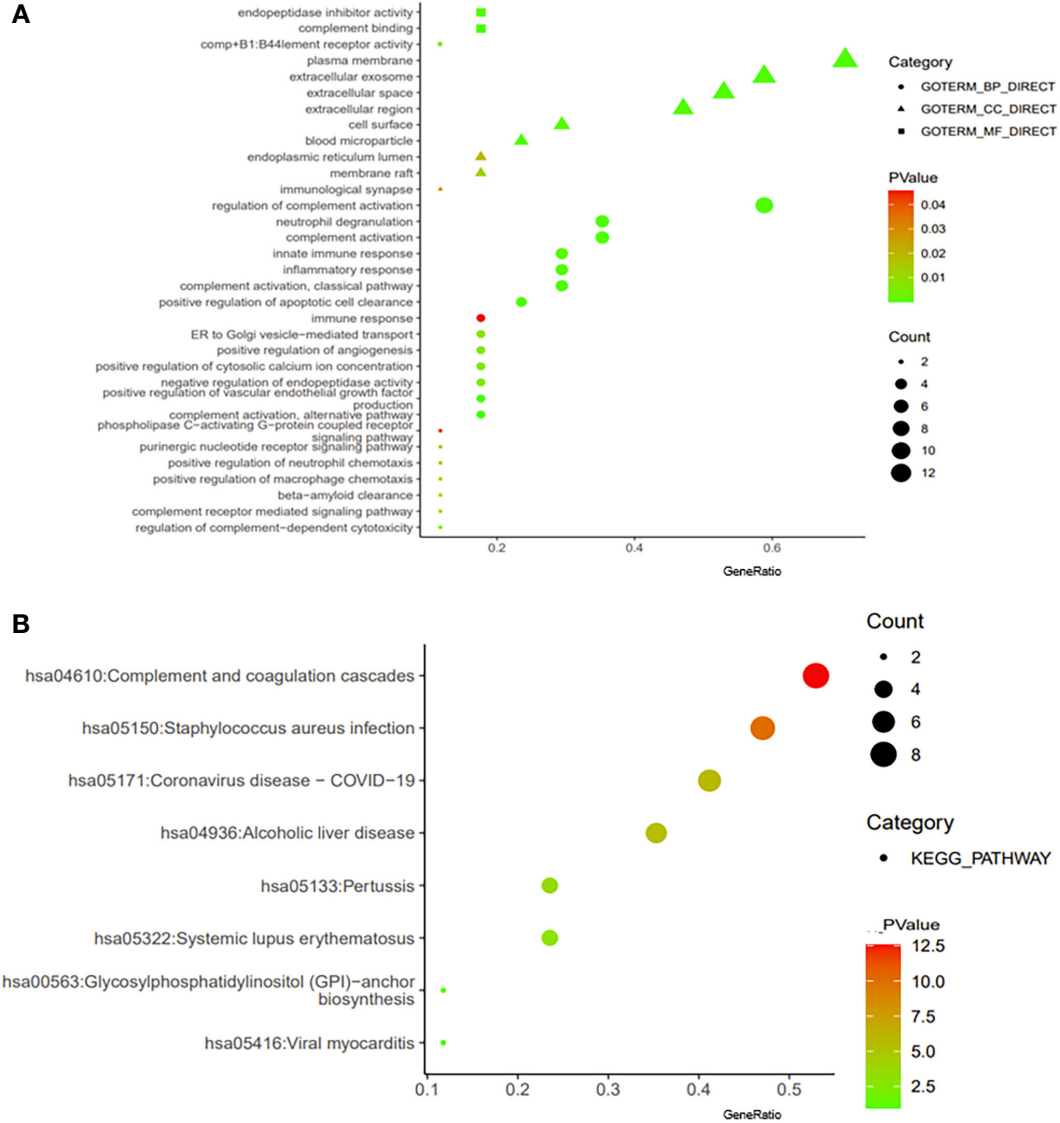
Enrichment analysis of *CD55*. The visualization of GO enrichment analysis **(A)** and KEGG enrichment analysis **(B)** of *CD55* by DAVID 6.8. GO, Gene Ontology; KEGG, Kyoto Encyclopedia of Genes and Genomes.

We constructed a PPI regulatory network for *CD55-*related proteins using the STRING database. When setting the size cutoff to “no more than 20 interactors”, *CFP*, *CFB*, *C4A*, *C4B*, *C5AR1*, *C3*, *C3AR1*, *C2*, *EGF*, *LCK*, *CD59*, *PIGA*, *PGAP1*, *ICAM1*, *EMR2*, and *CD97* proteins were incorporated (**Figure 6**). The top three GO enrichment involved in complement activation and apoptotic cell clearance at the biological process level was as follows: extracellular exosome, extracellular space, and plasma membrane at the cellular component level, and complement binding, endopeptidase inhibitor activity, and complement receptor activity at the molecular function level (**Figure 7A**; **Table 2**). KEGG pathway enrichment analysis showed that these genes were significantly enriched in complement and coagulation cascade pathways, *Staphylococcus aureus* infection pathways, coronavirus disease–COVID-19, alcoholic liver disease pathways, pertussis pathways, etc. (**Figure 7B**; **Table 3**).

**Table 2:** GO pathway enrichment analysis.

GO	ID	Description	*p*-Value	Gene ID
BP	GO:0030449	Regulation of complement activation	1.02E−17	*C4B*, *C3*, *C4A*, *C5AR1*, *C3AR1*, *CD59*, *CFP*, *CD55*, *CFB*, *C2*
BP	GO:006956	Complement activation	4.96E−09	*C4B*, *C3*, *C4A*, *CFP*, *CFB*, *C2*
BP	GO:2000427	Positive regulation of apoptotic cell clearance	6.02E−09	*C4B*, *C3*, *C4A*, *C2*
CC	GO:0070062	Extracellular exosome	2.76E−06	*C4B*, *C3*, *C4A*, *LCK*, *EGF*, *CD59*, *CD55*, *CFB*, *ICAM1*, *C2*
CC	GO:0005615	Extracellular space	1.14E−05	*C4B*, *C3*, *C4A*, *EGF*, *CD59*, *CFP*, *CFB*, *ICAM1*, *C2*
CC	GO:0005886	Plasma membrane	3.35E−05	*C4B*, *C3*, *C4A*, *LCK*, *EGF*, *C5AR1*, *C3AR1*, *CD59*, *CFP*, *CD55*, *CFB*, *ICAM1*
MF	GO:0001848	Complement binding	7.74E−06	*C4B*, *CD59*, *CFB*
MF	GO:0004866	Endopeptidase inhibitor activity	4.80E−04	*C4B*, *C3*, *C4A*
MF	GO:0004875	Complement receptor activity	0.01	*C5AR1*, *C3AR1*

GO, Gene Ontology; BP, biological process; MF, molecular function; CC, cellular component.

**Table 3:** KEGG pathway enrichment analysis.

ID	Description	*p*-Value	Gene ID
hsa04610	Complement and coagulation cascades	2.96E−13	*C4B*, *C3*, *C4A*, *C5AR1*, *C3AR1*, *CD59*, *CD55*, *CFB*, *C2*
hsa05150	*Staphylococcus aureus* infection	8.36E−11	*C4B*, *C3*, *C4A*, *C5AR1*, *C3AR1*, *CFB*, *ICAM1*, *C2*
hsa05171	Coronavirus disease–COVID-19	1.27E−06	*C4B*, *C3*, *C4A*, *C5AR1*, *C3AR1*, *CFB*, *C2*
hsa04936	Alcoholic liver disease	2.70E−06	*C4B*, *C3*, *C4A*, *C5AR1*, *C3AR1*, *C2*
hsa05133	Pertussis	2.67E−04	*C4B*, *C3*, *C4A*, *C2*

KEGG, Kyoto Encyclopedia of Genes and Genomes.

## 4 Discussion

The complement system in humans remains on standby and constantly on high alert for any potential intruders (15). The membrane complement regulatory proteins (*CD35*, *CD46*, *CD55*, and *CD59*) are important regulators of the complement system and are widely expressed on the surface of cells. In addition to the normal cells and tissues, the mCRPs also protect malignant cells from complement attack (16). For example, all hematopoietic cells, as well as endothelial and epithelial tissues, express *CD55*/*DAF* (17, 18). In comparison to the surrounding normal tissues, *CD55*/*DAF* is often expressed at substantially higher levels in a variety of malignant cells, including cells of CRC (19). The findings of the present study are consistent with those of the previous studies.

According to the UALCAN program, CRC tissues had higher levels of *CD55* than normal tissue, which is supported by the data from the IHC and HPA databases (20). Notably, high expression of *CD55* can also promote the dissemination of tumor cells in circulation. Blocking or downregulating *CD55* may be an important step in advancing the efficacy of monoclonal antibody (mAb) immunotherapy for cancer (16).

*Transcription factor CP2* (*TFCP2*) belongs to the *TFCP2/Grainyhead* family. The ubiquitous expression of *TFCP2* suggests its involvement in comprehensive cellular functions and diseases such as cancer, Alzheimer’s disease, and AIDS (21). Previous studies have identified *TFCP2* as a pro-cancer factor in hepatocellular carcinoma (22), pancreatic cancer (23), breast cancer (24), and CRC (25). Additionally, *TFCP2* acts as a tumor suppressor, inhibiting the development of melanoma (26). Using the TRANSFAC online program, we found that *TFCP2* potentially binds to the promoter of *CD55*. To verify this, we conducted a dual-luciferase reporter assay. After knocking down *TFCP2* by siRNA, we found that the promoter activity of *CD55* reduced by 21%; however, after the mutated *TFCP2* binding site, it reduced by 66%. RNA interference (RNAi) is a natural process of target mRNA degradation. After we used si-*TFCP2*, the expression *TFCP2* was decreased by more than 50%; however, *TFCP2* could still bind to the promoter of *CD55*. After we mutated the binding site of *TFCP2*, *TFCP2* hardly binds to the promoter of *CD55* to regulate the expression of *CD55*. We then performed a ChIP assay and found that *TFCP2* directly binds to the promoter of *CD55* in colon cancer cells.

*Nuclear factor-kappa B* (*NF-κB*), belonging to the *Rel/NF-κB* family, regulates innate and adaptive immune responses (27). *NF-κB* is involved in tumorigenesis by regulating some important cell cycle-related genes, promoting cell proliferation, and inhibiting cell death (28). Using the TRANSFAC program, we found that *NF-κB* might bind to the promoter of *CD55*, which was subsequently confirmed by the dual-luciferase reporter assay. Colorectal cancer is a typical inflammation-dependent cancer (29, 30). *NF-κB* signaling is shown to link inflammation and the development of cancer (31). Moreover, ChIP provided clear evidence to support this finding.

MiRNAs are small non-coding regulatory RNAs (ncRNAs) that inhibit gene expression at a post-transcriptional level by binding to the 3′UTR of target mRNAs (32). Minor changes in miRNA have significant effects on gene expression (33). The dysregulation of miRNA was found in colon cancer tissues (34). Studies have shown that miR-27a-3p targets *BTG1* to affect the biological phenotype of colorectal and ovarian cancer cells (35, 36). Additionally, in colon cancer cells, wild-type *p53* downregulated the expression of miR-27a-3p (37). In the current study, through TargetScanHuman 7.2, we predicted that miR-27a-3p targeted the 3′UTR of *CD55*. Subsequently, *CD55* was confirmed as a direct target of miR-27a-3p by a dual-luciferase reporter gene assay. It is reasonable to speculate that miR-27a-3p affects colon cancer cells by targeting *CD55*.

DNA methylation is a form of epigenetic modification. Aberrant DNA methylation has been identified as an important aspect of promoting CRC pathogenesis by silencing tumor suppressor genes. Studies have found that DNA hypermethylation could promote CRC metastasis by regulating *CEBPB* and *TFCP2* (38). Using the SurvivalMeth program, we found that the promoter region of *CD55* does have hypermethylation sites in colon cancer tissues. However, we did not find any overlap between hypomethylation sites in colorectal cancer cells and any of the putative binding sites identified on the *CD55* promoter or impaired *NF-κB* binding to the *CD55* promoter. Future research is still required to identify the mechanism.

The PPI network showed that *CD55* and the key complement system components *C3*, *C4A*, and *C4B* are closely related to colon cancer. Studies have shown that C3 is also an immune-related core differential protein in colon adenocarcinoma (COAD) and is negatively correlated with the overall survival of COAD patients (39). Recent studies proposed that deficiency or pharmacological blockade of *C5aR1* significantly impeded tumorigenesis of CRC, and the over-expression of *C5aR1* contributed to the poor prognosis of CRC patients (40, 41). *CFP* plays a positive role in regulating the natural immune system in alternative pathways, and it is associated with immune infiltration in gastric cancer and lung cancer (42). We speculated that *CFP* and *CD55* might be involved in the immune infiltration of tumor cells in colon cancer. This evidence indicated that *CD55* promoted cancer by regulating complement activation either directly or indirectly (43).

In conclusion, the expression of *CD55* in colon cancer was associated with the genetic regulation of *TFCP2*, *NF-κB*, epigenetic regulation of miR-27a-3p, and methylation modification. The genes associated with *CD55* are probably involved in immune-related pathways in colon cancer. This study provides a theoretical basis and insight into the development of biomarkers for future research in the field of colon cancer.

## Data availability statement

The datasets presented in this study can be found in online repositories. The names of the repository/repositories and accession number(s) can be found in the article/supplementary material.

## Author contributions

JL and NF: conceptualization, writing—original draft, and validation. ZY, AL, HW, YJ, HX, and QS: methodology and validation. AL and SJ: software and data curation. ZZ and XZ: supervision, writing—review and editing, and project administration. XZ: funding acquisition. All authors contributed to the article and approved the submitted version.
